# Evaluation of Types of Vertigo With Electronystagmography: An Experience From a Tertiary Care Hospital in West Bengal, India

**DOI:** 10.7759/cureus.35496

**Published:** 2023-02-26

**Authors:** Soumik Saha, Atish Haldar, Himel Mondal

**Affiliations:** 1 Otorhinolaryngology, Tamralipto Government Medical College and Hospital, Tamluk, IND; 2 Otorhinolaryngology, Deben Mahata Government Medical College and Hospital, Purulia, IND; 3 Physiology, All India Institute of Medical Sciences, Deoghar, IND

**Keywords:** muscle, labyrinth, syncope, otorhinolaryngologic diseases, cerebellum, brain stem, inner ear, vertigo, vestibular function tests, electronystagmography

## Abstract

Background

Electronystagmography (ENG) is a diagnostic test that measures the electrical activity of the muscles that control eye movements. ENG has the potential to identify the cause of vertigo by assessing the function of the vestibular system. Vertigo can be of two types - peripheral or central. In addition, a combination of peripheral and central types may coexist. Peripheral vertigo is caused by pathology in the inner ear and central vertigo is caused by pathology in the brainstem or cerebellum.

Objective

This study aimed to evaluate the applicability of ENG in assisting the diagnosis of the type of vertigo in a remote tertiary care center in West Bengal, India.

Materials and methods

This cross-sectional study was conducted in tertiary care hospital in West Bengal, India. Any patient presenting first time with a complaint of vertigo was approached and recruited for the study after taking written informed consent. We collected demographics and conducted a complete ear, nose, and throat examination, including otoscopy and audiological evaluation. A consensus between two expert otorhinolaryngologists was reached for the categorization of vertigo. Then, ENG was performed to assess the vestibular function to help aid the categorization. Magnetic resonance imaging (MRI) and computed tomography (CT) scans were done in central vertigo patients according to the necessity to diagnose the cause. Data were presented in descriptive statistical terms and categorical data were compared by Chi-square test.

Result

A total of 84 patients (male 31, female 53) with a median age of 25 years (Q1-Q3: 21-30.25) participated in the study. We found 75% of the patients were complaining of instability, 50% rotatory objective vertigo, 29.76% falling tendency, 22.62% blackout, and 2.38% sinking sensation. The majority of the patients (63%) had two or more symptoms. A total of 68 (80.95%) patients could be categorized into peripheral (46 [54.76%]) and central (22 [26.19%]) types. When we added ENG to the tests, we could categorize all the patients and found that 48 (57.14%) had peripheral, 27 (32.14%) had central, and nine (10.71%) had mixed lesions.

Conclusion

ENG when used in conjunction with clinical examination, otoscopy, and an audiological examination can help to categorize all patients into peripheral, central, or mixed lesion types of vertigo. Hence, ENG can be an important tool in identifying the type of vertigo and can aid in appropriate treatment decisions.

## Introduction

Vertigo is a common and debilitating symptom affecting millions of people worldwide, with a prevalence of 0.71% in the Indian rural population [1]. Vertigo is a sensation of spinning or whirling, which is often accompanied by dizziness, nausea, and vomiting. According to the etiology, vertigo is divided into two categories - peripheral and central [2]. Peripheral vertigo is caused by pathology in the inner ear, which is responsible for maintaining balance and spatial orientation. It is typically characterized by sudden, intense episodes of dizziness, often accompanied by nausea and vomiting. Central vertigo is caused by pathology in the brainstem or cerebellum, which is responsible for processing sensory information and controlling balance. It is often characterized by a more gradual onset of symptoms and may be associated with other neurological symptoms, such as double vision or difficulty speaking [3].

Vertigo is commonly diagnosed through a combination of detailed medical history, general physical examination, examination of the ear and associated organs, and diagnostic tests. The diagnosis is made based on the characteristic symptoms of vertigo, such as a feeling of spinning or dizziness, and the exclusion of other possible underlying causes. During a physical examination, the doctor may look for signs of nystagmus, which is an involuntary movement of the eyes that can occur with vertigo [4]. The doctor may also test balance and coordination by asking the patient to perform certain movements or tasks. In addition to the physical examination, several diagnostic tests may be used to help to diagnose vertigo, including audiometry, electronystagmography (ENG), videonystagmography, magnetic resonance imaging (MRI), and computerized tomography (CT) scan [5].

The diagnosis and management of vertigo are complex and challenging due to the heterogeneity of its underlying causes. ENG is a widely used diagnostic test that measures the electrical activity of the muscles that control eye movements. It can provide valuable information about the function of the vestibular system and help diagnose different types of vertigo. ENG is a non-invasive and safe test that can be performed in an outpatient setting [6]. In rural areas where facilities for advanced imaging like MRI or CT scan are not available, ENG can help to aid provisional diagnosis. No previous study was conducted in this region to establish the utility of the ENG for categorizing the type of vertigo.

This study aims to evaluate the types of vertigo using ENG in a tertiary care hospital in West Bengal, India. The study will provide insights into the prevalence of different types of vertigo and their underlying causes in this region, which can help improve the diagnosis and management of vertigo. The results of this study will also contribute to the existing literature on the use of ENG in the evaluation of vertigo.

## Materials and methods

Study design and setting

This cross-sectional observational study was conducted in the Department of Otolaryngology of Tamralipta Government Medical College and Hospital, Tamluk, West Bengal, India. This is a tertiary care teaching hospital in West Bengal, India. The study duration was from January to December 2022. The study was approved by the Institutional Review Board (TGMCH/IRB/22/01-1). Written informed consent was obtained from all the research participants. Any patient whose age was below 18 years, provided assent for participation and full written informed consent was signed by parents or legal guardians.

Study participants

We recruited the patients as study participants from the outpatient department of the Department of Otolaryngology. This was a convenience type of sampling. Any patient presenting with vertigo for the first time was approached and recruited after getting consent. A total of 92 patients presented with vertigo during this period and 87 of them consented to participate. A total of three patients were excluded due to previous history of surgery on the ear. We used a convenience sampling technique for recruiting the research participants with inclusion and exclusion criteria. Patients of any age and gender coming with complaints of vertigo first time in the outpatient department and providing written consent for participation were included in the study. Any patient with any medication for vertigo, had a history of surgeries like mastoidectomy or stapes surgery, suffered from chronic otitis media, hypertension or diabetes mellitus, or any psychiatric disorder was excluded from the study.

Data collection

**Figure 1:**
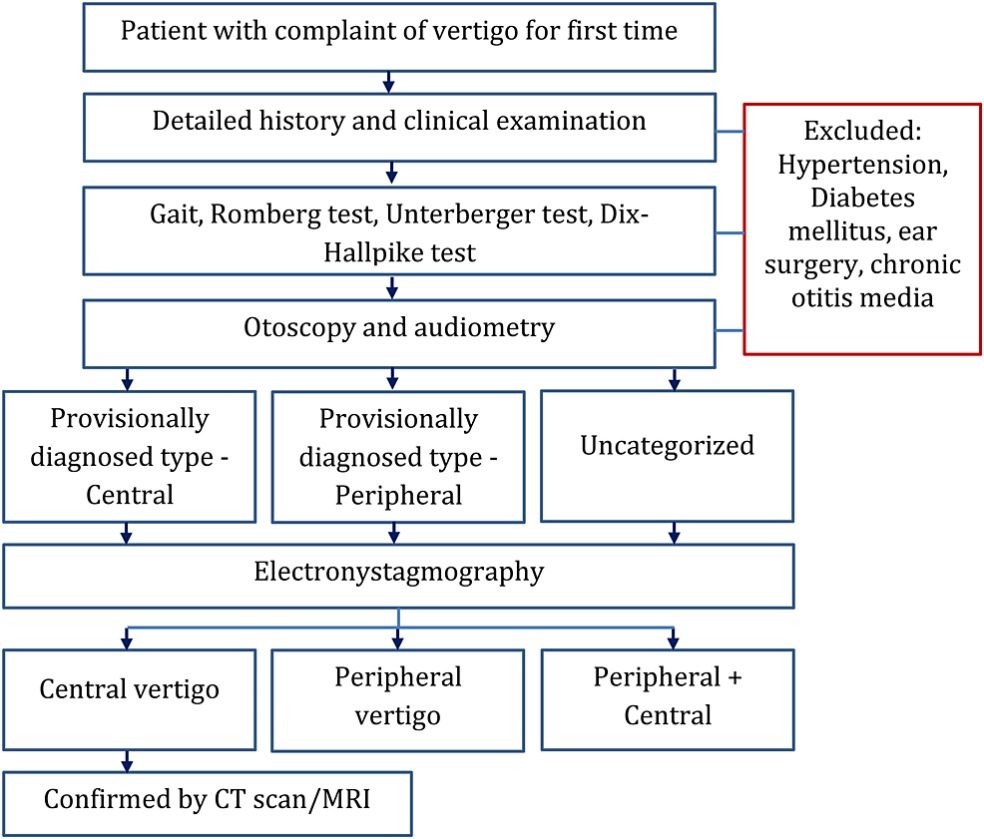
Brief study procedure flow chart CT: Computed tomography, MRI: Magnetic resonance imaging

We collected demographic information, medical history, and frequency of vertigo symptoms in a structured questionnaire. Clinical examination for vertigo - gait, Romberg test, Unterberge test, and Dix-Hallpike test was done. All participants underwent a complete ear, nose, and throat examination, including otoscopy (for checking external ear and tympanic membrane) and audiological evaluation (for the sensorineural hearing disorder) by two expert otorhinolaryngologists having an experience of >10 years. A consensus was reached for categorizing the patients into a peripheral or central type of vertigo, and patients were kept uncategorized where a provisional diagnosis was not possible. Then, ENG was performed using GLADIUS Window-based ENG (Recorders and Medicare Systems Private Ltd, Budanpur, India) to assess the vestibular function. All ENG procedures were performed by a trained technician. The technician was not aware of the provisional diagnosis. The participants were instructed to sit comfortably in a dark room and recording electrodes were placed on the forehead and around the eyes according to the device manual. After calibration, ENG was performed for spontenous nystagmus with closed eyes, to 30 degrees left and right, pendular tracking, positional and optokinetic test, caloric test with irrigating the ear. The ENG finding was used in addition to the previous test reports to categorize the patients. After the categorization of patients, those who were in the central type were undergone CT/MRI according to necessity for confirmation of the pathology. The flow chart of the study is presented in Figure 1.

Data analysis

The data were analyzed using GraphPad Prism 7 (GraphPad Software Inc., San Diego, CA, USA). Descriptive statistics were used to summarize the demographic and clinical characteristics of the participants. Categorical variables were compared by the Chi-square test. A p-value of less than 0.05 was considered statistically significant.

## Results

A total of 84 patients (male 31, female 53) with a median age of 25 years (Q1-Q3: 21-30.25) participated in the study. Age-wise distribution of research participants and other demographic descriptions is shown in Table 1.

**Table 1:** Number of patients according to age, sex, residence, marital, and social status Social status is calculated according to the modified Kuppuswamy scale

Parameter	Category	Values	p-value
Sex	Male	31	0.02
	Female	53	
Age (years)	11-20	15	<0.0001
	21-30	48	
	31-40	11	
	41-50	8	
	51-60	2	
Residence	Urban	6	<0.0001
	Semi urban	26	
	Rural	52	
Marital status	Unmarried	47	<0.0001
	Married	33	
	Divorced/Widow/Widower	4	
Social status	Upper	2	<0.0001
	Upper middle	4	
	Lower middle	30	
	Upper lower	21	
	Lower	27	

**Figure 2:**
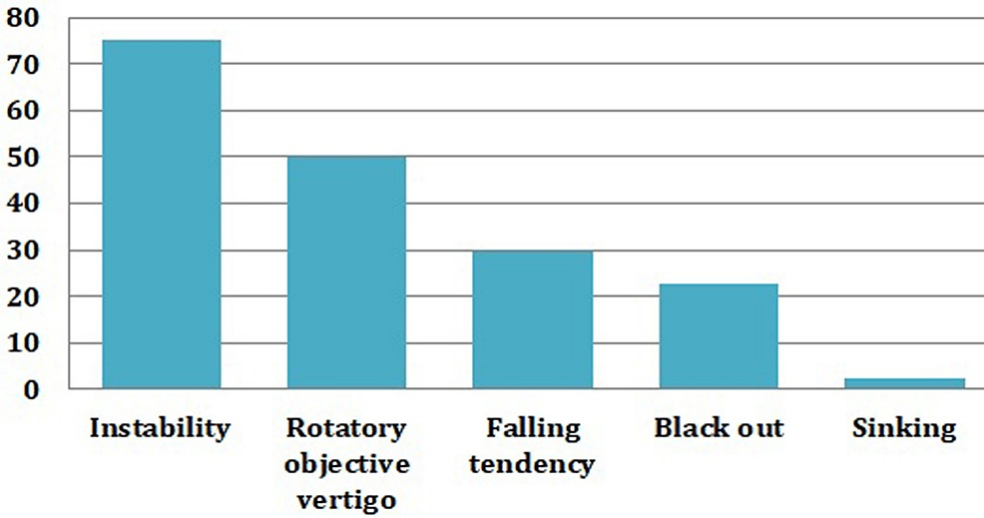
Percentage of patients according to symptoms Chi-square test p-value <0.0001

Clinical symptoms-wise patient distribution is shown in Figure 2. The distribution is shown according to the percentage of symptoms. The total percentage would not make a total of 100 as more than one symptom was present in many of the patients. We found that 75% of the patients were having instability followed by rotatory objective vertigo (50%).

Categories of vertigo without and with ENG are shown in Table 2. A total of 16 patients could not be categorized without ENG. However, when ENG was combined with other tests, all the patients could be categorized. The CT scan/MRI done in the central and mixed type of vertigo showed pathology of the central type in all cases.

**Table 2:** Number of patients according to categories of vertigo Chi-square (3) = 25.55, p-value <0.0001 The presence of both peripheral and mixed types of lesions is categorized as "mixed" ENG: Electronystagmography

Category of vertigo	Without ENG	With ENG
Central	22	27
Peripheral	46	48
Mixed	0	9
Uncategorized	16	0

**Figure 3:**
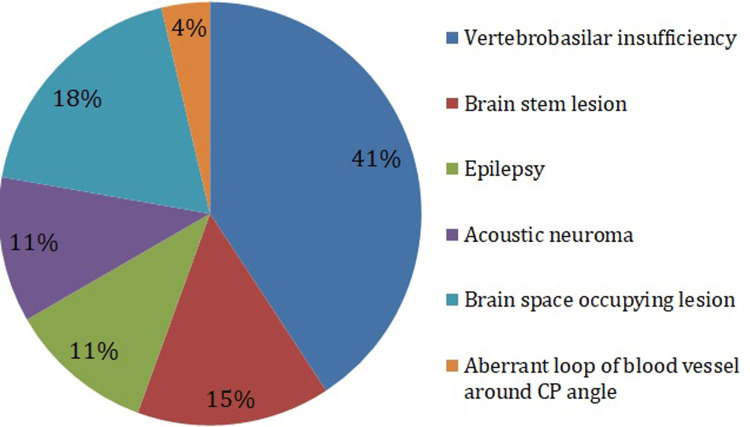
Causes of central vertigo CP: Cerebellopontine Chi-square p-value = 0.02 (a significant p-value indicates that the causes are not distributed by chance)

We found in this study that the most common cause of central vertigo is vertibro-basillar insufficiency (41%) followed by brain stem lesion (15%). The causes observed in this study are shown in Figure 3.

**Figure 4:**
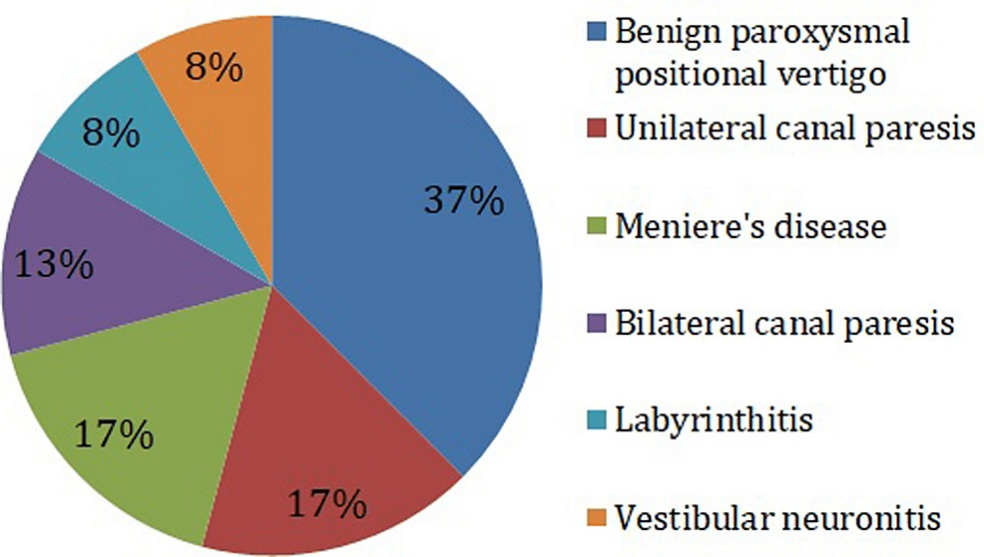
Causes of peripheral vertigo Chi-square p-value = 0.005 (a significant p-value indicates that the causes are not distributed by chance)

The most common type of peripheral vertigo was benign paroxysmal positional vertigo (37%) followed by unilateral canal paresis and Meniere's disease with 17% each. The percentage of the peripheral lesions observed in this study is shown in Figure 4.

In mixed type, we found four cases with benign paroxysmal positional vertigo and epilepsy, two cases with canal paresis and epilepsy, two cases with vertebrobasilar insufficiency and labyrinthitis, and one case of vertebrobasilar insufficiency with benign paroxysmal positional vertigo.

We observed 15 patterns of ENG Butterfly Trinary code as shown in Table 3. The most common type was “0000” (35.71%) followed by “0010” (11.9%).

**Table 3:** Observed patterns of ENG Butterfly Trinary code ENG: Electronystagmography

Trinary code	Number of cases	Percentage
0000	30	35.71
0001	6	7.14
2020	4	4.76
0020	4	4.76
1011	2	2.38
0222	2	2.38
1010	2	2.38
0210	2	2.38
0010	10	11.9
1111	4	4.76
0111	4	4.76
1000	4	4.76
1100	4	4.76
1110	2	2.38
0011	4	4.76

## Discussion

With an aim to find the applicability of ENG in finding the cause of vertigo and categorizing it as central, peripheral, or mixed type, we found that when ENG is added to the other clinical and audiological tests, the patients could be categorized successfully. Without adding the ENG, approximately 19% of patients could not be categorized in our study. This finding is corroborative of the study conducted by Gupta and Mundra [7]. The value of ENG in the diagnosis of vertigo is high, as it can help to provide important diagnostic information about the category of vertigo that can guide further investigations and clinical decision-making [8]. However, it should be noted that ENG is just one of several diagnostic tools that may be used in the evaluation of vertigo, and its use should be determined based on the specific needs of each patient.

While ENG can be a valuable diagnostic tool for evaluating vertigo, there are some potential disadvantages to consider as well [9]. ENG involves attaching electrodes to the skin around the eyes, which may be uncomfortable for some patients. In addition, the caloric test, which involves irrigating the ear with water or air, can cause dizziness and nausea. In some cases, ENG may detect abnormalities in eye movements that are not related to a vestibular disorder. This can lead to unnecessary further testing or treatment. In addition, ENG may fail to detect a vestibular disorder in some patients [10]. This can be due to a variety of factors, including the patient's physiology or the timing of the test. In many settings in developing countries, ENG can be a relatively expensive and time-consuming diagnostic test, which may limit its availability to some patients or healthcare settings [11]. However, an ENG has the potential to reduce the necessity of an MRI/CT scan [12]. Another limitation of ENG is that it requires specialized equipment and trained personnel to administer and interpret the results. In some cases, technical difficulties or user errors may affect the accuracy or reliability of the test.

CT and MRI scans can be helpful in certain cases of vertigo; they are not always necessary or appropriate for every patient. A healthcare provider can help determine whether imaging tests are warranted based on the individual patient's symptoms, medical history, and physical exam findings along with ENG. Limitations of imaging techniques in the diagnosis of vertigo include lack of sensitivity, limited availability, higher cost, and potential risk [13,14]. Hence ENG plays a vital role in the diagnosis and management of these patients.

Limitations

This study has some limitations that should be considered when interpreting the results. The study was conducted in a single tertiary care hospital in a particular state of India, which may not be representative of the general population of the country. The sample size was relatively small, which may limit the generalizability of the findings. The diagnosis and categorization were done by humans and subjective components may be there. ENG is a subjective test that may be influenced by several factors, such as the technician's experience and the participant's level of cooperation.

## Conclusions

ENG is a useful noninvasive diagnostic tool for categorizing the type of vertigo in patients. The study found that ENG when used in conjunction with clinical examination, otoscopy, and audiological examination has potential to categorize all patients into peripheral, central, or mixed lesion types of vertigo. Hence, ENG can be an important tool in identifying the type of vertigo and can aid in appropriate treatment decisions. Furthermore, the use of ENG has the potential to avoid MRI or CT scans. However, this should be evaluated in further studies.

## Appendices

Contribution details

Dr. Soumik Saha: Concept, Administration, Data collection, Literature search, Editing manuscript

Dr. Atish Haldar: Concept, Literature search, Editing manuscript

Dr. Himel Mondal: Data analysis, Data visualization, Literature search, Drafting the manuscript
